# Decellularized Macroalgae as Complex Hydrophilic Structures for Skin Tissue Engineering and Drug Delivery

**DOI:** 10.3390/gels10110704

**Published:** 2024-10-31

**Authors:** Andreea Luca, Florina-Daniela Cojocaru, Maria Stella Pascal, Teodora Vlad, Isabella Nacu, Catalina Anisoara Peptu, Maria Butnaru, Liliana Verestiuc

**Affiliations:** 1 Department of Biomedical Sciences, Faculty of Medical Bioengineering, “Grigore T. Popa” University of Medicine and Pharmacy, 700115 Iasi, Romania; andreea.luca@umfiasi.ro (A.L.); florina.cojocaru@umfiasi.ro (F.-D.C.); bim-rom-1934@students.umfiasi.ro (M.S.P.); bim-rom-1956@students.umfiasi.ro (T.V.); nacu.isabella@gmail.com (I.N.); maria.butnaru@umfiasi.ro (M.B.); 2 “Petru Poni” Institute of Macromolecular Chemistry, 41 A Grigore Ghica Voda Alley, 700487 Iasi, Romania; 3 Cristofor Simionescu Faculty of Chemical Engineering and Environmental Protection, Gheorghe Asachi Technical University of Iaşi, 700050 Iasi, Romania; catipeptu@tuiasi.ro

**Keywords:** hydrogels, tissue engineering, drug delivery, macroalgae, decellularization

## Abstract

Due to their indisputable biocompatibility and abundant source, biopolymers are widely used to prepare hydrogels for skin tissue engineering. Among them, cellulose is a great option for this challenging application due to its increased water retention capacity, mechanical strength, versatility and unlimited availability. Since algae are an unexploited source of cellulose, the novelty of this study is the decellularization of two different species, freshly collected from the Black Sea coast, using two different chemical surfactants (sodium dodecyl sulphate and Triton X-100), and characterisation of the resulted complex biopolymeric 3D matrices. The algae nature and decellularization agent significantly influenced the matrices porosity, while the values obtained for the hydration degree included them in hydrogel class. Moreover, their capacity to retain and then controllably release an anti-inflammatory drug, ibuprofen, led us to recommend the obtained structures as drug delivery systems. The decellularized macroalgae hydrogels are bioadhesive and cytocompatible in direct contact with human keratinocytes and represent a great support for cells. Finally, it was noticed that human keratinocytes (HaCaT cell line) adhered and populated the structures during a monitoring period of 14 days.

## 1. Introduction

Tissue engineering complexity resides from an interdependent use of matrices and cells designed as combinations that mimic biological structures properties and functions. In general, the matrices used in tissue engineering applications have the role to substitute the extremely diverse extracellular matrix functions, providing mechanical support, cell-anchorage cues, determining cell phenotype and differentiation, influencing proliferation and cell growth [1,2].

For skin tissue engineering, in particular, hydrogels are among the most used materials due to their good adherence to wounds or other cutaneous defects, long resistance and ability to function as efficient drug delivery systems (DDS) [3]. DDS are complex therapeutically approaches that gained significant attention in the last decade, coming as a solution for the multiple drawbacks and limitations of conventional drug formulations: reduced half-time (which involves frequent administration), poor bioavailability, and decreased solubility [4].

Various techniques and molecules were considered for the designing and synthesis of complex architectures, such as hydrogels for tissue engineering and drug delivery applications. These materials provide appropriate hydrophilicity and versatile response to some physical, chemical, or biological stimuli. However, there is still a real challenge in achieving proper biocompatibility and cell–biomaterial interactions; therefore, researchers turned their attention towards another source, insufficiently explored as biomaterials and environmental friendly: plant tissues [5,6,7].

Cellulose is a linear polysaccharide, with different origins: plants (most abundantly), bacteria, or algae. Cellulose main function is to offer mechanical stability and the possibility of vertical growth, together with other accompanying components, such as lignin or hemicellulose [8]. Tissue engineering applications have tried to exploit the variability of cellulose characteristics depending on its source: differences in cellulose crystallinity degree (bacterial cellulose presents higher crystallinity), porosity resulting after lyophilization (higher porosity for plant-based cellulose compared to bacterial), or nanofibers production ability (ribbon-like structure in the case of bacterial cellulose) [9]. Regarding biomedical applications, due to its biocompatibility, water retention capacity, and versatility when combined with other polymers and mechanical properties, cellulose has been used for wound dressing, drug delivery, and bone and cartilage tissue engineering [10,11,12,13].

An approachable method to produce cellulose-based structures, for various biomedical applications, includes the decellularization of vegetal tissue [14]. Thereby, decellularized apple was studied in adipose tissue engineering for inducing osteogenesis and probiotic encapsulation [15,16,17]; spinach leaves were studied for ascorbic acid release as a stimulator for fibroblasts [18], cabbage for bone tissue engineering [19], leatherleaf for small-diameter vascular grafts [20], celery for tendon tissue engineering [21], decellularized mushroom tissue for myogenic differentiation [22], and seaweed for fibroblasts cell growth [14]. The diversity of applications shows a great potential of cellulose obtained through the decellularization of vegetal tissue.

A less-explored source of cellulose is represented by macroalgae, so far studied mainly for their use in nutrition and pharmaceutical field and less for their potential as biomaterials [23]. It is already known that green macroalgae contain a high percentage of sulphates and carbohydrates in their composition and also include vitamins, flavonoids, phenolic compounds, and oligosaccharides. As pharmacological compounds, the extracts from macroalgae present antiviral, antibacterial, or antifungal activity [24,25,26,27].

Macroalgae were used for polysaccharide extraction, with the resulting macromolecules representing important components for obtaining versatile biomaterials and also as drug delivery systems [25]. Macroalgae cell wall structure depends on the type of algae and is mainly composed of cellulose microfibrils and ulvan in the case of green macroalgae, and of cellulose microfibrils in a different type of arrangement forming a network with glucomannan, sulphated glucan, and sulphated xylogalactans in the case of red algae. The average cellulose content was determined to be 9.67% for green algae and 4.75% for red algae [28,29]. Other polysaccharides isolated from various macroalgae classes are alginate, fucoidan, laminarin (all three from brown algae), carrageenan, and agar (red algae) [30]. After extracting the above-mentioned biomolecules and removing the cellular components, the remaining structure is mainly based on cellulose, representing a biocompatible hydrophilic matrix that can sustain cells attachment and proliferation, suitable for applications like wound dressing and controlled drug delivery. Other remarkable advantages of this matrix reside from avoiding any ethical and antigenicity concerns, specific to animal-derived extracellular matrix and from replacing the complexity of laboratory synthesis [28,29].

Regarding nutrition, Cadar et al. presented the use of green algae biocompounds, harvested from the Black Sea, as nutraceuticals, based on their antimicrobial and antioxidant properties [23]. Although Black Sea provides a significant macroalgae source, the research mentioned above is one of the few data reported.

For the preparation of skin tissue engineering hydrogels, cellulose is mainly used after several chemical modifications [31,32,33], resulting in artificial scaffolds. On the other hand, decellularization will bring in front natural scaffolds, with superior biological properties, sustaining proper cellular interactions, degradability, and mimicking tissue microenvironment [34]. For this reason, the aim of this study was to use decellularized macroalgae matrices, mainly based on cellulose (according to the investigated chemical composition), as hydrogels for drug delivery and skin tissue engineering applications. Two different types of macroalgae (green algae—*Ulva lactuca* and red algae—*Rhodophyta*), were decellularized and lyophilized, and their morphology was analysed using stereomicroscopy, inversed microscopy and scanning electron microscopy (SEM). Since the application of the matrices is for skin tissue engineering, other important properties were further analysed, such as the hydration degree, as well as bioadhesive properties and capacity to incorporate and controllably release drugs. Finally, the cytocompatibility of the decellularized matrices and their ability to allow cell adherence on their surfaces were tested, evaluating the differences between the two types of algae when populated with human keratinocytes. This study highlights some original data, and others very few or not yet reported: the valorisation of macroalgae from Black Sea as biomaterials, an unexploited source of cellulose (macroalgae), and a rare but valuable technique used to obtain complex matrices for skin tissue engineering and drug delivery from vegetal tissue (decellularization).

## 2. Results and Discussion

Decellularization has emerged as an important technique in tissue engineering, starting from animal tissue and evolving to whole-organ decellularization in an attempt to respond to the urgent need of donors for various diseases. For certain domains, like skin tissue engineering, decellularization offered acellular dermal matrices isolated from animal tissue, used in the clinical practice [35], but proved to have serious drawbacks related to ethics, cost, or safety [36]. Therefore, attention was drawn towards plant tissue decellularization, by cellular content removal and preservation of the cell walls composed mainly of cellulose. Cellulose can provide a plentiful resource with numerous properties necessary in tissue engineering applications and wound dressing, like biocompatibility, hydrophilic behaviour (hydrogels), mechanical resistance, and ease of manipulation [37,38].

As a result of plant decellularization, the cellulosic hydrogel structures present interconnected pores that facilitate cell integration, nutrient and water exchange, and waste removal.

### 2.1. Macroalgae Decellularization

The decellularization of the green and red macroalgae was performed using two agents, ionic SDS and non-ionic Triton X-100, in order to remove the cell content with the preservation of the 3D hydrophilic matrix. The obtained vegetal structures decellularized with SDS were codified as red algae SDS, respectively, and green algae SDS, while those decellularized with Triton X-100 were codified as red algae Triton and green algae Triton. During the decellularization process, a faster evolution was observed for the macroalgae immersed in SDS, compared to those immersed in Triton. The matrices became translucent as an indicator of biological compounds’ removal and were further investigated for their chemical structure and morphology.

### 2.2. Chemical Structure and Morphology of the Decellularized Macroalgae

**Figure 1:**
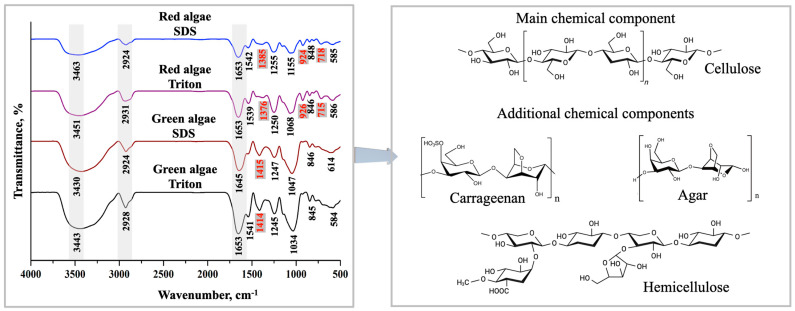
Chemical structure of the decellularized macroalgae (FTIR).

FTIR is a valuable technique for the chemical composition analysis of plant specimens by offering precise details about samples isolated from the same species, as well as samples from different species (leaf structure, composition variation, and so on) [39]. The FTIR spectra for the decellularized structures are shown in Figure 1.

At first glance, evident differences, in terms of chemical structure, between the two macroalgae species can be remarked in the region of 500 cm^−1^ and 1600 cm^−1^. In the case of red algae, two supplementary peaks, at 715–718 cm^−1^ (C–O–C bending vibrations in glycosidic linkages) and 924–926 cm^−1^, were observed, attributed to C–O–C vibrations of the 3,6-anhydro-a-_L_-galactopyranose unit, also found in agar and carrageenan [40,41]. Another variation was the presence of peak 1376–1385 cm^−1^ for red algae, instead of 1414–1415 cm^−1^ in the case of green algae. The region 1376–1385 cm^−1^ corresponded to the ester sulphate (–S=O) compound, while 1414–1415 cm^−1^ corresponded to the C–O bond and marked the presence of free –COOH groups [41,42]. A further remark is related to the type of surfactant used, which had not influenced, in an obvious way, the chemical structure of the obtained matrices.

For the same region analysed above (500–1600 cm^−1^), matrices from both macroalgae species were characterised by low-density bands at 845–848 cm^−1^ that could be assigned to galactose-4-sulphate unit, the bands 1034–1086 cm^−1^ to the skeleton of galactans, and the band at 1115 cm^−1^ to the cellulose C–O bond [41,42,43]. The bands 1245–1255 cm^−1^ appeared due to sulphate ester groups (–S=O) identified in agar or due to stretching vibration mode of the CO–OR (acyl-oxygen) identified in hemicellulose [44,45]. The last peaks highlighted at 1539, 1541, and 1542 cm^−1^ were also found by Khan et al. when studying algal biomass and, according to them, represent the amide-II absorption of the protein [46].

In the region 1600–4000 cm^−1^, three important peaks were recognized for all the four structures. The first one, around 1653 cm^−1^, represented the –OH bending vibration of the adsorbed H_2_O specific to cellulosic materials [45]. Looking further on the spectra, the prominent bands between 2924 and 2931 cm^−1^ represented C–H stretching vibration, and those from 3430 to 3463 cm^−1^ represented O–H stretching, defining cellulose [47].

To summarize FTIR results, the decellularization process had not modified the chemical structure of the biopolymeric skeleton of macroalgae, mainly based on cellulose, hemicellulose, carrageenan, and agar.

Black Sea-harvested Ulva lactuca’s chemical composition was studied by Cadar et al. and Negreanu-Pîrjol et al., revealing a complex physico-chemical composition, with an insoluble fibre content of 32% and a carbohydrate content of 55% for green algae, compared to 51.9% carbohydrate content for the red species [23,48]. For the green macroalgae the insoluble fibre content was represented by 20.6% hemicellulose, 9.13% cellulose, and 1.56% lignin, according to Yaich Y. et al. [49]. The insoluble fibre content for red algae was reported to be between 12 and 16%, lower than for the green Ulva lactuca [50]. After the decellularization process, the remaining structure of the macroalgae was also evaluated using haematoxylin eosin staining in order to microscopically observe the absence of the nuclei on the plant cells and to better highlight the cellulosic cell walls. This type of staining uses haematoxylin for colouring nuclear components in blue, while eosin is used for staining in pink cytoplasmic components. In Figure S1 from the Supplementary Materials, the lack of blue staining can be observed, indicating the absence of the cell nuclei as a result of the decellularization, confirming the aspect of the macroalgae that was observed macroscopically.

**Figure 2:**
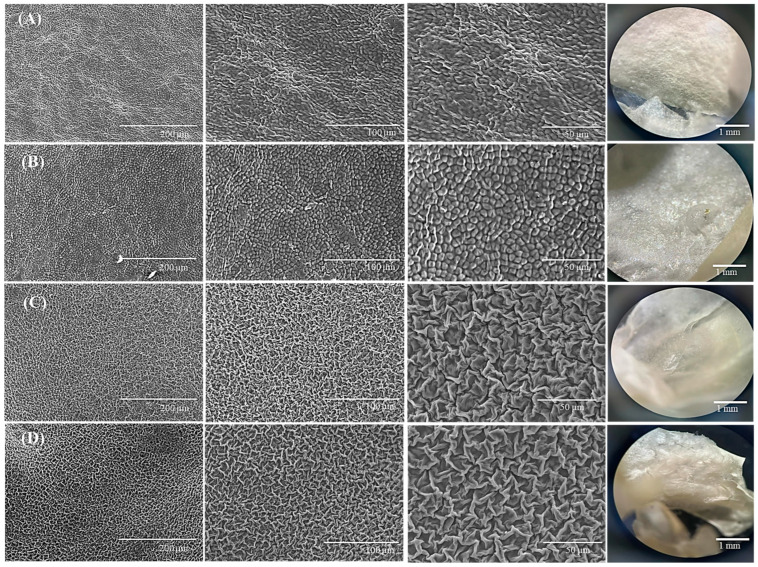
Morphology of the decellularized macroalgae: (**A**)—red algae SDS; (**B**)—red algae Triton; (**C**)—green algae SDS; (**D**)—green algae Triton (SEM and stereomicroscope images—last column).

Moreover, the morphology of the decellularized structures was analysed by stereomicroscopy and SEM (Figure 2), revealing differences between the two types of algae: the green algae present an open-pore structure, while for red algae, the remaining cellulose gave a compact, closed-pore appearance.

For the red macroalgae, the cell wall is mainly composed of a crystalline phase, containing cellulose and other polysaccharides (inner wall) and an amorphous phase, particularly based on agar and carrageenan (exterior wall). Due to their hydrophilicity, agar and carrageenan are mostly removed during the decellularization process [51]. The removal of some polysaccharides can determine internal collapsing of the cell walls, explaining the closed-pore structure observed in the SEM images. On the other hand, green macroalgae cell walls are an intricate matrix of hydrophilic cellulose, ulvan fibres, xyloglucan, and glucuronan, bond by hydrogen linkages and ionic interactions [52]. Analysing Figure 2, the visible morphology variations, observed for the two types of decellularized structures, could be associated with the aforementioned differences in composition and also with cellulose microfibrils arrangement: linear for green macroalgae, and matrix-like for red macroalgae [29]. The pore size and pore size distribution of the decellularized macroalgae was analysed using Image J software, version 1.54g. According to the data in the Table 1, green algae-based matrices had pores with sizes that were influenced by the surfactant used for the decellularization process: the anionic surfactant, SDS, led to obtaining larger pores in the case of green algae compared to the non-ionic Triton. The image processing for the red algae supports did not highlight pores, confirming the compact, closed-pore architecture.

### 2.3. Hydration Degree of the Decellularized Macroalgae

The hydration degree is an important aspect for a scaffold intended to be used in tissue engineering applications, since it influences the absorption of nutrients from the media and waste exchange, ensuring support for normal cell development. In this case, cellulose represents an optimal candidate based on its hydrophilicity and possibility to interact with the negatively charged cell surface [14].

As it can be seen in Table 1, the hydration degree (HD) was significantly influenced by the agent used in the decellularization process, since the values obtained for SDS treated macroalgae were between 1500 and 2000 (1654% green algae SDS, 1869% red algae SDS), and for Triton treated macroalgae, they were around 3000% (3033% green algae SDS, 2954% red algae Triton). This strong hydrophilic character was attributed to the polar groups of the polymeric network, highlighted by FTIR: –OH, –SO_3_H, and –COOH [53].

According to Waseeq Ur Rehman et al., if a material can absorb and retain more than 90% aqueous solutions, it is defined as a hydrogel [54]. Their capacity to swallow in water and maintain a 3D structure is similar to natural living tissues, making it possible to recommend them for important biomedical applications: contact lenses, tissue engineering, and drug delivery systems [55]. The data obtained for this assay suggest that all decellularized macroalgae structures can be included in hydrogel class.

### 2.4. Drug Loading and In Vitro Drug Release

Ibuprofen is often used as a drug model in a significant number of skin engineering applications, mainly due to its anti-inflammatory effects [56,57,58]. The mechanism that leads to the analgesic effect is associated with a non-selective inhibition of specific enzymes, cyclooxygenase, which participate to the arachidonic acid conversion in prostaglandin and mediate inflammation and pain [59]. Loading ibuprofen in skin tissue engineering constructs (based on polymers) will reduce one of its main side effects: multiple oral administrations due to its short half-life; in addition, patients’ comfort will be improved [56].

**Figure 3:**
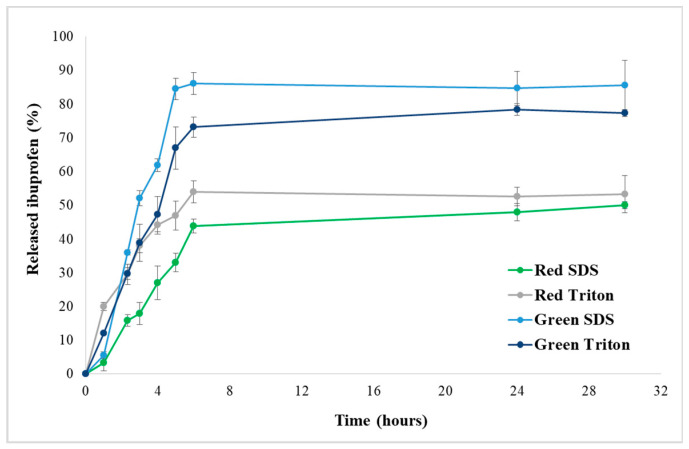
Ibuprofen release from hydrophilic decellularized macroalgae.

Ibuprofen has a slightly water solubility and a high solubility in most organic solvents, including methanol and ethanol [60]. Therefore, the drug loading was performed from low concentrated water solutions and calculated as 10% (wt:wt) reported to biopolymeric hydrophilic matrix. The drug loaded was evaluated spectrophotometrically by measuring the concentration of drug in the loading solution. Different values were calculated for the tested algae: 71 ± 2 µg/mg for green algae—SDS, 66 ± 5 µg/mg for green algae—Triton, 62 ± 4 µg/mg for red algae—SDS, and 56 ± 3 µg/mg for red algae—Triton. The drug loading capacity was associated with scaffold porosity and its components’ interaction with Ibuprofen molecules: a larger porosity and ionic surfactant contributed to a higher content of drug in matrices. The in vitro release studies were performed in PBS, at 37 °C, simulating the physiological conditions in the human body. In order to obtain the drug release profiles, the cumulative release was plotted versus time, and the kinetic curves are depicted in Figure 3.

The release profiles for the decellularized macroalgae hydrogels indicated a steady, controlled release of ibuprofen over 4–6 h, with a maximum of release varying from 45% for red algae SDS to 87% for green algae SDS. The drug was gradually released in the first time intervals and then reached a constant release. The possible reasons include the surface adsorption, specific surface area, and scaffolds’ morphology. The drug loading and release profile depend on the decellularized macroalgae structure and morphology, the architectures’ ability to absorb and retain Ibuprofen solution, and the drug–matrix interaction. The Ibuprofen molecule can be involved in various molecular associations due to its amphiphilic characteristics. Hydrogen bonds between the COOH group from drug and the hydrophilic moiety from polysaccharides, generally present in all hydrogels, are responsible for the drug attachment to the 3D polymeric network; meanwhile, the morphology regulate the drug free diffusion. A good correlation with matrices morphology (pores distribution and interconnections) and swelling degree was also noticed: green algae SDS presented a higher porosity and ability to swell and a faster release of Ibuprofen. Generally, the drug located on the surface of the matrix is easily released due to labile interactions with polysaccharide chains.

It is well known that several phenomena are governing hydrogels drug release: diffusion, network relaxation, erosion, depending on hydrogel composition, network crosslinking degree and flexibility, as well as the drug structure, molecular volume, and interactions with polymeric matrix [61]. In order to explain the drug release mechanism, the obtained data were fitted with Korsmeyer–Peppas model, using Equation (1) [62]:

(1) $$\frac{M_{t}}{M_{\infty }}=k\times t^{n}$$

where M_t_ = amount of drug released at time “t”; M_∞_ = total amount of drug in the decellularized matrix; k = release rate constant (Table 1); n = diffusion exponent; t = time (h).

The diffusional exponent ‘n’ indicates the mechanism of drug release and is matrix composition- and geometry-dependent. A Fickian diffusion is produced for n < 0.45, and diffusion is the predominant process; when 0.45 < n < 0.89, the drug transport is non-Fickian (anomalous), and a balance between diffusion and relaxation rates is registered; n > 0.89 is associated with the Case II mechanism of transport, and polymer relaxation is considered determinant [63]. The values obtained for ‘n’ (Table 1) suggest a Fickian transport, indicating that Ibuprofen was released from all decellularized hydrogels by diffusion and no erosion of the matrix was produced. Matrix relaxation contribution was not essential for the drug transport, indicating a stable and limited elastic 3D architecture. The changes in the hydrogel matrix morphology from a porous organization in the case of green algae-based support to a denser architecture for red algae-based support reduced the cellulose relaxation, thereby influencing the Ibuprofen release mechanism.

### 2.5. In Vitro Bioadhesion of the Decellularized Macroalgae

Effective bioadhesion involves the adherence of the material to the tissue surface mediated mainly by intermolecular blocking [64]. The surface of the biomaterial will influence the interactions with cells: a surface that will allow a flattened cell shape, as opposed to a spherical one, suggests a good cell–biomaterial interaction [65], being an important property of the biomaterials used in skin tissue engineering applications. Cellulose presents a good cell adhesion mechanism due to its hydrophilic groups [37].

The bioadhesion mechanism is deeply correlated with materials hydrophilicity and ability to absorb water or biological fluids. Generally, the residence time on the wounds is influenced by the presence of exudates, proteins, enzymes, and other molecules involved in the regeneration mechanism. Continuously, some molecules are secreted, migrate, and are renewed [66]. The hydrogel swelling influences the entanglement and interpenetration of the polymeric chain with macromolecules from wound area, and after that, hydrogen bonds and electrostatic bonds will connect the material with tissue.

**Figure 4:**
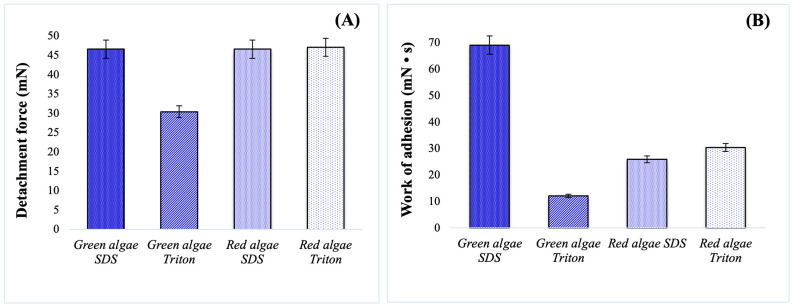
Bioadhesion properties of the decellularized macroalgae: (**A**) detachment force; (**B**) work of adhesion.

Figure 4 shows the results obtained for in vitro bioadhesion studies, represented by the force of detachment of the decellularized macroalgae from a simulated biological membrane and the work of adhesion.

For red algae, the decellularization reagent did not influence the detachment force, and comparable results with green algae SDS were obtained (Figure 4A). An interesting observation was related to Triton treatment on green macroalgae, where both detachment force and work of adhesion were significantly reduced. The behaviour was associated with the lower swelling properties and limited content of soluble polysaccharides, hemicellulose, carrageenan, and agar. Another explanation can be related with the fact that ulvan, a sulphated polysaccharide identified in green algae, plays an important role in adhesion mechanisms and also helps water retention [67,68]. Further, as already known, the porosity of a matrix for skin tissue engineering will strongly influence cell and tissue adhesion, and Figure 2 highlights an open-pore structure only for green algae [69]. As mentioned before, the results can be correlated with the retention degree (Table 1), since the maximum values were obtained for SDS decellularized macroalgae. In the case of green algae, the variations were more evident, with the %HD of green algae SDS being almost double compared with green algae Triton, while for red algae, the difference was smaller.

### 2.6. Cytocompatibility of the Decellularized Macroalgae

**Figure 5:**
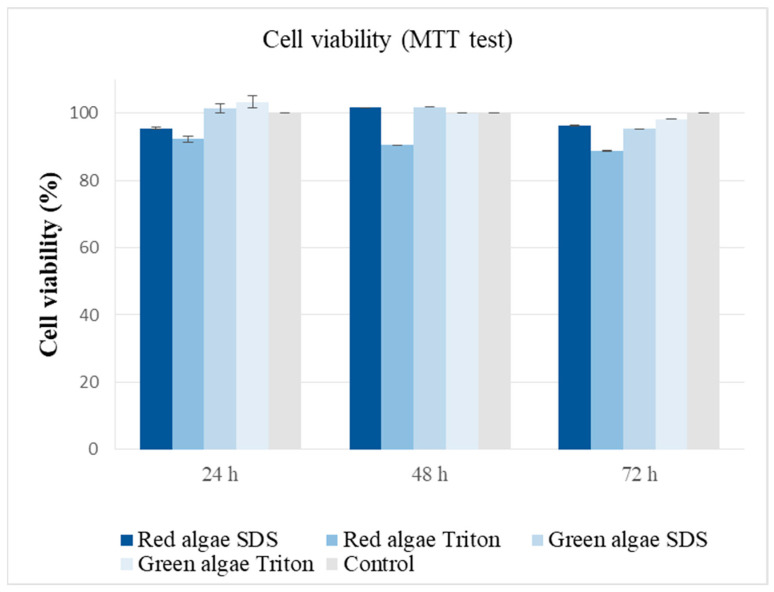
Cell viability after 24, 48, and 72 h of HaCaT cell culturing with the decellularized macroalgae.

The decellularized macroalgae matrices obtained in this study had a 3D hydrophilic network with hydrogel features, being promising materials for skin tissue engineering applications. Therefore, their cytocompatibility was tested on HaCaT cell line using the MTT test (Figure 5).

The results indicate the cytocompatibility of the obtained structures and a complete removal of the decellularization agents during the purification phase, as it can be observed in Figure 5, where cell viability values, obtained at three different intervals (24 h, 48 h, and 72 h), compared to control, were over 90%.

**Figure 6:**
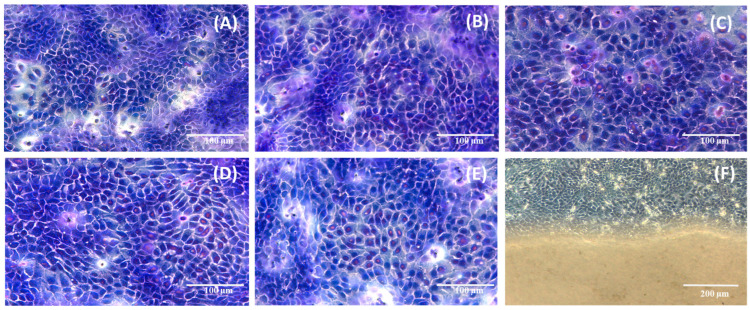
HaCaT cells morphology after 7 days contact with decellularized macroalgae: (**A**) control; (**B**) red algae SDS; (**C**) red algae Triton; (**D**) green algae SDS; (**E**) green algae Triton; (**F**) cultured cells and algae surface.

An important indicator of the effects that decellularized macroalgae might have on cells was the observation of cell morphology after 7 days exposure compared to control, using May–Grünwald and Giemsa staining. In Figure 6, it can be observed that cell morphology remained unmodified compared to the control and also that cells continued to proliferate, as no differences were seen between the cells cultured in the presence of decellularized macroalgae matrices and control. Also, no differences were observed between red and green macroalgae, irrespective of the used decellularization agent.

**Figure 7:**
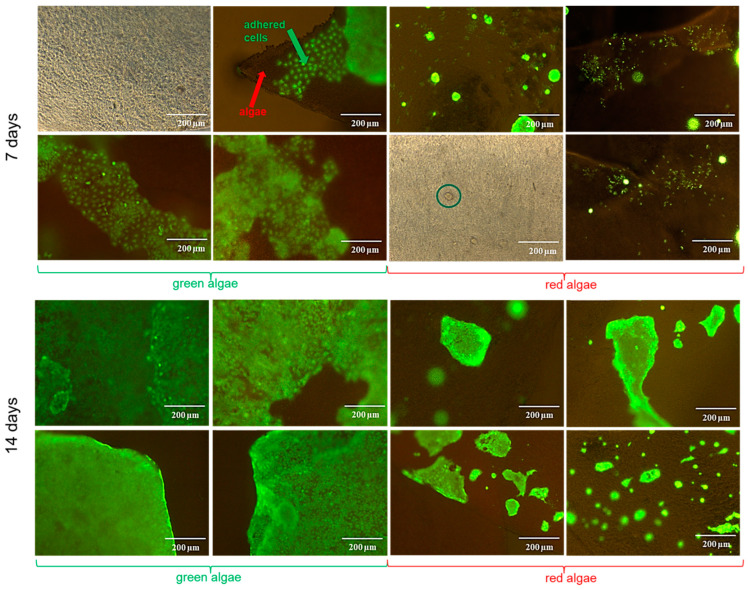
Cell population of the decellularized green and red macroalgae after 7 and 14 days of culture, calcein–AM-stained and brightfield images. The green circle indicates a cluster of cells onto the red algae after 7 days of culture.

Considering the obtained results for the interaction with HaCaT cells, we studied the ability of the decellularized macroalgae to be populated with cells. For this, cell suspension was placed onto the surface of the algae matrices, left to adhere, and then followed in culture for 14 days. After 7 days, calcein was used to examine cell adherence. In Figure 7, it a difference regarding the cell-adherence properties of the two macroalgae can be observed: for the green ones, cells were beginning to cover the surface, presenting an elongated morphology and forming a monolayer, while for the red algae, the cells had a tendency to develop in isolated groups scattered throughout the surface, with a less elongated morphology.

This behaviour was maintained at day 14, als, being observed that, in the case of green algae, the entire surface was covered with a layer of cells, resembling a tissue, while for the red algae, the isolated groups expanded, but the cell distribution was irregular, and the cells tended to cluster, regardless of the decellularization agent used. Green algae porous structure evidenced by SEM images (Figure 2) promoted better cell adherence and proliferation. However, the differences in cell behaviour between the two types of algae could have also been influenced by a different alignment and concentration of cellulose microfibrils. It was shown that depending on the group of algae, α-cellulose will predominate in the algae containing agar and alginate and β-cellulose in those groups containing carrageenan. Furthermore, the dominance of α cellulose is related with a higher thermodynamic reactivity, a great advantage of algae-derived cellulose, compared with plant-derived cellulose [70]. Green algae, particularly of the Chlorophyta class, which includes the Ulvales order, present, on average, higher cellulose content and a higher yield of α cellulose compared to red algae of the Rhodophyta class [71]. Another particular element of the Ulva species is ulvan composition, containing both soluble (20.53%) and insoluble fibres (34.27%) [47,72]. This polysaccharide contains rhamnose, which is recognized by human keratinocytes lectins, acting as a possible receptor by mediating intracellular transmission pathways and gene expression. In fibroblasts, this interaction determines an increased calcium influx with a stimulating effect on cell proliferation [47,73,74]. In their native environment, cells adhere to the extracellular matrix (ECM) due to an interaction between adhesion receptors found on cell surface and ECM proteins. When biomaterials are used, this interaction has to be assured by the biomaterial’s surface properties, and in the case of cellulose, various derivatives were obtained to improve cell adhesion [75]. Regarding macroalgae cellulose, cell adherence onto its surface was obtained without cellulose chemical modification, consistent with a previous study, where NIH-3T3 cells were successfully cultured on porous and fibrous seaweeds matrices [14]. The differences between cell adhesion behaviours on the two decellularized macroalgae, green and red, can be explained by the different surface morphologies indicated by SEM analysis, as a porous surface like the one presented by green macroalgae will facilitate the adherence of cells [65]. Reactive hydroxyl groups found on the cellulose fibre are shown to influence cell adhesion due to their ability to immobilize proteins that will facilitate cell anchorage [76]. Different exposure of these groups revealed by FTIR analysis for the two types of decellularized macroalgae can also provide an explanation on the different cell adhesion patterns.

## 3. Conclusions

The present study highlights the possibility to obtain skin tissue engineering hydrogels from algae, an unlimited marine source, using a very simple method which involves only surfactants. The decellularized structures proved to be complex biopolymeric networks, with FTIR revealing absorptions specific to cellulose, hemicellulose, carrageenan, or agar. Regarding in vitro bioadhesion properties, green algae SDS matrices presented a higher work of adhesion in correlation with their superior hydrophilicity. Their hydration degree and ability to controllably release Ibuprofen sustained their use as drug delivery systems for skin tissue engineering. Moreover, green algae seemed to be more adequate than red algae, since human keratinocytes uniformly populated their structures, similar to a tissue. This phenomenon was strongly correlated with the open-pore structure of green algae, while red algae had a closed-pore structure and, as a result, the cells formed isolated groups.

As perspectives, the decellularized matrices loaded with ibuprofen will be also studied in direct contact with keratinocytes, and inflammatory biomarkers will be determined by enzyme-linked immunosorbent assay. Moreover, wound healing is also an assay planned, since it offers valuable information about skin tissue engineering structures in vitro.

## 4. Materials and Methods

### 4.1. Materials

Green (*Ulva lactuca*) and red macroalgae (*Rhodophyta*) were harvested fresh from the Romanian coast of the Black Sea in March 2024. For their decellularization, sodium dodecyl sulfate (SDS) and Triton X-100 (Triton) were used in a 1% concentration (both purchased from Sigma Aldrich, Darmstadt, Germany). Type 3 ultrapure water from a system (Arium^®^ Mini, Goettingen, Germany) was used to prepare all the solutions and in the purification steps. Dulbecco’s Modified Eagle’s Medium/Nutrient Mixture F-12 Ham (DMEM F12/Ham); Hanks’ Balanced Salt Solution (HBSS); foetal bovine serum (FBS); a mixture of the following antibiotics: 5000 units of penicillin, 5 mg/mL streptomycin, and 10 mg/mL neomycin; 3-(4,5-dimethylthiazol-2-yl)-2,5-diphenyltetrazolium (MTT); phosphate-buffered saline tablets (PBS—one tablet mixed with 200 mL water, resulted in a PBS solution pH = 7.4, 0.01 M); dimethylsulfoxide (DMSO); calcein–AM solution in DMSO (4 mM); May–Grünwald stain and Giemsa stain; absolute ethanol; and formaldehyde were also provided by Sigma-Aldrich, Darmstadt, Germany. Human epidermal keratinocytes (HaCaT cell line) were purchased from AddexBio, San Diego, CA, USA.

### 4.2. Macroalgae Decellularization Process

**Figure 8:**
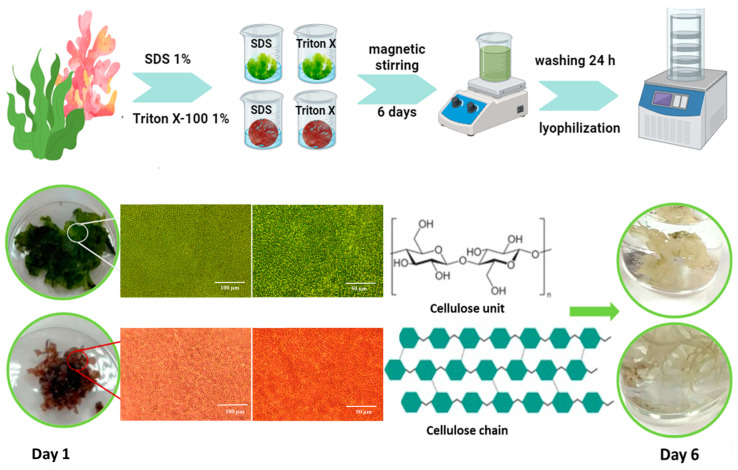
Decellularization process (created with BioRender.com, accessed on 1 August 2024). Macroscopic and microscopic aspect of macroalgae before (day 1) and after (day 6) decellularization.

Green and red algae were thoroughly washed to eliminate all the impurities and then immersed into separate recipients with SDS 1% and Triton X-100 1%, respectively, to observe the differences in the decellularization efficiency of the two agents (Figure 8). Then, 1 g of macroalgae was weighted and added in 100 mL surfactant solution used for decellularization (Triton X-100 1% and SDS 1%, respectively). The decellularization process was carried under permanent magnetic stirring for 6 days, with daily renewal of the decellularization agent (on the first day, there was two times/day renewal). After 6 days, the surfactants were removed, replaced with type 3 ultrapure water, and left for another 24 h, with three times/day water change to assure complete removal of the surfactants. The decellularized macroalgae were frozen at −20 °C overnight, and then were lyophilized at −55 °C, 0.2 mBar, for 12 h (a benchtop freeze dryer from Labconco, Kansas City, MO, USA was used).

### 4.3. Evaluation of the Chemical Structure and Morphology of the Decellularized Macroalgae

The chemical structure of decellularized macroalgae was studied by Fourier Transform Infrared Spectroscopy (FTIR), using Bruker Vertex 70 spectrophotometer (Bruker, Berlin, Germany) was used and the tests were performed on lyophilized samples, with a transmittance setting programmed at 400–4000 cm^−1^.

After decellularization and lyophilization, the morphology of the resulted matrices were first analysed using a stereomicroscope—Optika SFK-91D (Optika, Ponteranica, Italy) and an inversed microscope—Leica DMI3000 (Leica Microsystems GmbH, Wetzlar, Germany) along with their supporting software. The morphology was evaluated depending on the type of algae, and the decellularization process was assessed taking into consideration the surfactant efficiency, to confirm the removal of the biological material and the preservation of the cell walls. For a deeper perspective, the morphology and porosity of the decellularized samples was assessed with a HITACHI SU 1510 scanning electron microscope—SEM (Hitachi Company, Tokyo, Japan). The samples were evaluated in lyophilized form, after being mounted on an aluminum stub, fixed and coated with a 7 nm gold layer using a Cressington 108 Sputter Coater (Cressington Scientific Instruments Ltd., Watford, UK). The pore size and pore size distribution for the decellularized structures were analysed using Image J (version 1.54g) plugin for porous scaffolds analysis [77]. The assessment implies the following steps: conversion of pixel size units to metric units; picture segmentation; application of a morphological filter to separate related pores; verification and extraction of data regarding the identified pores. The technique separates the SEM images to isolate cavity regions, facilitating the determination of each individual pore size.

### 4.4. Determining the Hydration Degree of the Decellularized Macroalgae

Swelling tests were performed gravimetrically on lyophilized decellularized matrices, hydrated for three days and then weighed. After that, the scaffolds were maintained at 37 °C and weighted at different time intervals, until equilibrium. When the weight of the algae remained unmodified, the drying process was complete, the weight was measured and the hydration degree was determined using the Equation (2):

(2) $$\mathrm{HD}\;(\%)\;=\frac{W_{\mathrm{wet}}-W_{\mathrm{dry}}}{W_{\mathrm{dry}}}\;\times \;100$$

where W_wet_ represents the weight of the hydrated samples, while W_dry_ represents the weight of the dried decellularized matrices.

### 4.5. Drug Loading Procedure and In Vitro Drug Release

Ibuprofen, an anti-inflammatory drug, was used as drug model and loaded by diffusion in the decellularized matrices. First, the decellularized structures were immersed in an aqueous ibuprofen solution (10% wt:wt, reported to matrix; 15 μg/mL concentration of the solution) for 24 h at 37 °C. The drug loaded was evaluated spectrophotometrically by measuring the concentration of drug in the loading solution. The matrices removed from the drug solution were carefully dabbed on a filter paper to absorb the liquid excess, immersed in 1 mL PBS solution (pH = 7.4, 0.01 M), and placed again at 37 °C. At specific periods, 2 μL of supernatant was extracted and analysed at 220 nm using DeNovix DS-11+ microvolume spectrophotometer (DeNovix Inc., Wilmington, DE, USA). The wavelength of 220 nm was chosen after spectrophotometrically recording the aqueous ibuprofen solution (15 μg/mL) between 200 and 800 nm, with the maximum absorption wavelength (λmax) being found at 220 nm [78]. The concentration of released ibuprofen was determined using a calibration curve at the same wavelength. For each decellularized structure, the assay was performed in triplicate, with the results being graphically displayed as the mean ± the standard deviation. The release pharmacokinetic parameters (k—release rate and *n*—diffusional coefficient) were developed using the Korshmayer–Peppas exponential [31].

### 4.6. In Vitro Bioadhesion Tests

In order to test decellularized matrices bioadhesion properties, a texture analyser, Ta.TX Plus^®^ (Stable Microsystems, Godalming, UK), was used. The working parameters used for performing the test were the pre-test speed (0.5 mm/s), test speed (0.1 mm/s), post-test speed (0.1 mm/s), applied force (0.5 g), returning distance (15 mm), and contact time (60 s). Samples from each type of algae were cut as disks (ϕ 8 mm, similar to the probe cylinder of the analyser) and fixed on a cylinder with double-adhesive paper. Then, the decellularized macroalgae were tested in contact with a simulating membrane (dialysis tubing cellulose membrane, average flat width 43 mm, typical molecular weight cut-off of 14,000 Da), prepared according to the producer protocol (Sigma Aldrich, Darmstadt, Germany), since it was already confirmed comparable results on cellulose membrane as with animal mucosa tissues [79]. The membrane was fixed on the static device, and 50 µL of PBS (pH 7.4, 0.01 M) were added to simulate biological conditions. The tests were performed under controlled temperature, at 37 °C. The probe cylinder with decellularized macroalgae was lowered until it contacted the simulating membrane and left for 60 s, then the separation from the membrane was recorded and displayed by a specific software (Exponent, version V.6.1.11.0), with the maximum detachment force and the work of adhesion being calculated. Each sample was tested using six replicates, and the results are reported as the mean ± the standard deviation.

### 4.7. Cytocompatibility Analisys of the Decellularized Macroalgae

ISO10993 recommendations were used for testing the cytocompatibility of the decellularized macroalgae on HaCaT cell line (primary epidermal keratinocytes normal human adult, from AddexBio, San Diego, CA, USA) [80] and approved by the Ethics Committee of the University of Medicine and Pharmacy “Grigore T. Popa” Iasi, Romania (21654/2021). The cells were cultured in a 75 cm^2^ flask, DMEM F12/Ham medium completed with 10% FBS, and 1% antibiotics mixture, with the medium changed every other day and maintained at 37 °C, 5% CO_2_, 96% relative humidity in an incubator (Memmert GmbH, Schwabach, Germany). Upon the formation of a monolayer, the adherent cells were dissociated by trypsinization, counted, and tested for viability with trypan blue. Next, 48-well plates were used for cell distribution, with 1 × 10^4^ cells/well, and cultured for 24 h. The next day, the medium was changed in each well, and equal amounts of weighted decellularized macroalgae were distributed in triplicate to each tested well. The macroalgae were sterilized by immersion in sterile ethanol 70% for 30 min and then washed thoroughly with HBSS until complete removal of the ethanol and left in culture medium until use. MTT test was performed after 24, 48, and 72 h of culture with the macroalgae. For this, the decellularized macroalgae and complete medium were removed and replaced with MTT solution, 5 mg/mL, prepared in DMEM F12/Ham without serum or antibiotics. This was left in incubation conditions for three hours, so that the viable cells could metabolize the reactive into formazan crystals that would be solubilized using DMSO. The absorbance of the resulting solutions was determined using an UV/Vis plate reader (Tecan Sunrise Plate Reader, Tecan Trading AG, Männedorf, Switzerland) at 570 nm. The viability of the cells was calculated using to Equation (3), with the control wells representing cells cultivated without macroalgae:

(3) $$\mathrm{Cell}\;\mathrm{viability}\;\left(\%\right)=\frac{\mathrm{Absorbance}\;\mathrm{sample}}{\mathrm{Absorbance}\;\mathrm{control}}\;\times \;100$$

### 4.8. Cell Morphology Evaluation

Concerning the effect decellularized macroalgae had on HaCaT cells’ morphology, the sterilized macroalgae were left in direct contact with the cells for 7 days in specific cell culture conditions. The morphology modifications that could appear were compared with control cells cultivated in the same conditions. After that period, the cells were fixed with formaldehyde and then stained using May–Grünwald and Giemsa stains. First, the algae were removed, then the cells were washed with PBS, and the first May–Grünwald stain was applied and left for three minutes, with the purpose of highlighting the cytoplasm of the cells in a bluish colour. After removing May–Grünwald, the second stain was applied, Giemsa, prepared with alkaline water, at a concentration of 5%. After 30 min Giemsa was removed, the cells were washed, and their morphology and components were observed at the inversed microscope—Leica DMI3000 (Leica Microsystems GmbH, Wetzlar, Germany)—compared to the control.

### 4.9. Decellularized Macroalgae Cell Population with Keratinocytes

Because the intended application of the decellularized macroalgae was skin tissue engineering, the focus was to observe if they have suitable properties for allowing cell adherence, growth, and proliferation on their structure. For this, decellularized sterile samples of 0.5 × 0.5 cm, in triplicate for each type of algae and used surfactant, previously left for 24 h in DMEM F12/Ham, were placed on the wells of a 24-well plate. Keratinocytes suspension was prepared after the trypsinization of a subconfluent culture of cells and counted. Each decellularized algae was covered with a suspension of 1 × 10^5^ cells, then placed and left for two hours to allow for cell adherence. Complete medium was added to each well and, after 24 h, it was changed with fresh one. After 72 h, the algae were moved to new wells to ensure that the growing cells were only those cultured onto the surface of the algae and not adhered onto the wells’ surface. Cell culture was followed for 14 days, with media renewal every two days. After 7 and 14 days, the media were removed and replaced with calcein–AM cell staining solution prepared in HBSS (2:1000 dilution), and fluorescent microscopy visualization was performed using a Leica DMI3000 microscope (Leica Microsystems GmbH, Wetzlar, Germany) was performed.

## Figures and Tables

**Table 1:** Hydration degree of the decellularized macroalgae, model constants, and R^2^ of Ibuprofen diffusion profile from hydrogels.

Type of Algae	Surfactant	Pore Size (µm)		HD (%)	Release Rate Constant (k)	Diffusion Exponent (n)	Correlation Coefficient (R^2^)
		Min	Max				
Green algae	SDS	14.3 ± 5.2	107.1 ± 7.5	3033 ± 72	0.145	0.447	0.9952
	Triton	10.2 ± 4.1	97.3 ± 8.7	1654 ± 23	0.213	0418	0.9881
Red algae	SDS	*	*	2954 ± 59	0.179	0.395	0.9933
	Triton	*	*	1869 ± 15	0.328	0.421	0.9897

* Undetectable.

## Data Availability

The original contributions presented in the study are included in the article/supplementary material, further inquiries can be directed to the corresponding author..

## Supplementary Materials

The following supporting information can be downloaded at: https://www.mdpi.com/article/10.3390/gels10110704/s1, Figure S1: Morphology of the decellularized macroalgae observed after haematoxylin and eosin staining (A—red algae SDS (20× 40×); B—red algae Triton; C—green algae SDS; D—green algae Triton).
