# Supplementary material for: Decellularized Macroalgae as Complex Hydrophilic Structures for Skin Tissue Engineering and Drug Delivery
Source: Gels. 2024 Oct 31;10(11):704. doi: 10.3390/gels10110704 (PMC11593777; doi:10.3390/gels10110704)
Supplement: Supplementary file 1 [file gels-10-00704-s001.zip › gels-3271658-supplementary.pdf]

Supplementary Materials

Decellularized Macroalgae as Complex Hydrophilic Structures for Skin Tissue Engineering and Drug Delivery

Andreea Luca [1], Florina-Daniela Cojocaru [1], Maria Stella Pascal [1], Teodora Vlad [1], Isabella Nacu [1,2], Catalina Anisoara Peptu [3], Maria Butnaru [1] and Liliana Verestiuc [1,*]

[1] Department of Biomedical Sciences, Faculty of Medical Bioengineering, "Grigore T. Popa" University of Medicine and Pharmacy, 700115 Iasi, Romania; andreea.luca@umfiasi.ro (A.L.); florina.cojocaru@umfiasi.ro (F.-D.C.); bim-rom-1934@students.umfiasi.ro (M.S.P.); bim-rom-1956@students.umfiasi.ro (T.V.); nacu.isabella@gmail.com (I.N.); maria.butnaru@umfiasi.ro (M.B.)

[2] "Petru Poni" Institute of Macromolecular Chemistry, 41 A Grigore Ghica Voda Alley, 700487 Iasi, Romania

[3] Cristofor Simionescu Faculty of Chemical Engineering and Environmental Protection, Gheorghe Asachi Technical University of Iaşi, 700050 Iași, Romania; catipeptu@tuiasi.ro

* Correspondence: liliana.verestiuc@bioinginerie.ro

Hematoxylin eosin staining of the decellularized macroalgae

Mayer's hemalum solution and Eosin Y-solution 0.5% aqueous (Merck KGaA, Dermstadt, Germany) were applied according to the producer's instructions on each of the decellularized macroalgae. After coloring, the sections were placed on glass slides, covered, and visualized at the inversed microscope—Leica DMI3000.

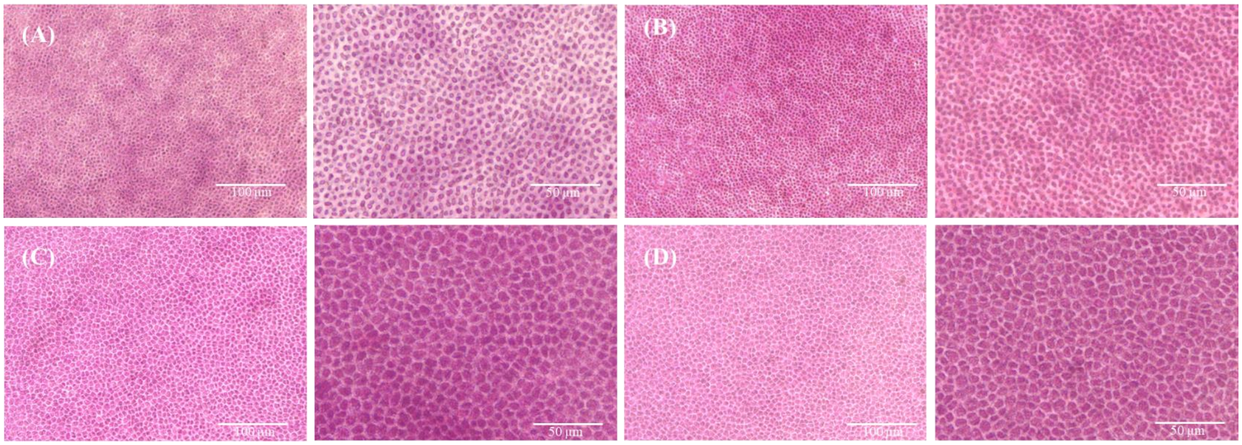

Figure S1. Morphology of the decellularized macroalgae observed after haematoxylin and eosin staining (A–red algae SDS (20×, 40×); B–red algae Triton; C–green algae SDS; D–green algae Triton)
